# Climate variation alters the synchrony of host–parasitoid interactions

**DOI:** 10.1002/ece3.3384

**Published:** 2017-09-14

**Authors:** Miles T. Wetherington, David E. Jennings, Paula M. Shrewsbury, Jian J. Duan

**Affiliations:** ^1^ Department of Entomology University of Maryland College Park MD USA; ^2^ Beneficial Insects Introduction Research Unit United States Department of Agriculture Agricultural Research Service Newark DE USA

**Keywords:** *Agrilus planipennis*, biological control, climate change, diapause, *Oobius agrili*, parasitism, phenology

## Abstract

Observed changes in mean temperature and increased frequency of extreme climate events have already impacted the distributions and phenologies of various organisms, including insects. Although some research has examined how parasitoids will respond to colder temperatures or experimental warming, we know relatively little about how increased variation in temperature and humidity could affect interactions between parasitoids and their hosts. Using a study system consisting of emerald ash borer (EAB), *Agrilus planipennis*, and its egg parasitoid *Oobius agrili*, we conducted environmentally controlled laboratory experiments to investigate how increased seasonal climate variation affected the synchrony of host–parasitoid interactions. We hypothesized that increased climate variation would lead to decreases in host and parasitoid survival, host fecundity, and percent parasitism (independent of host density), while also influencing percent diapause in parasitoids. EAB was reared in environmental chambers under four climate variation treatments (standard deviations in temperature of 1.24, 3.00, 3.60, and 4.79°C), while *O*. *agrili* experiments were conducted in the same environmental chambers using a 4 × 3 design (four climate variation treatments × 3 EAB egg densities). We found that EAB fecundity was negatively associated with temperature variation and that temperature variation altered the temporal egg laying distribution of EAB. Additionally, even moderate increases in temperature variation affected parasitoid emergence times, while decreasing percent parasitism and survival. Furthermore, percent diapause in parasitoids was positively associated with humidity variation. Our findings indicate that relatively small changes in the frequency and severity of extreme climate events have the potential to phenologically isolate emerging parasitoids from host eggs, which in the absence of alternative hosts could lead to localized extinctions. More broadly, these results indicate how climate change could affect various life history parameters in insects, and have implications for consumer–resource stability and biological control.

## 1 INTRODUCTION

Various climate models predict seasonal temperature and rainfall extremes to increase in severity and frequency over the coming century (Kharin, Zwiers, Zhang, & Hegerl, 2007; Yao et al. 2013; Wuebbles et al., 2014). The observed changes in mean temperature and increased frequency of extreme climate events have already impacted the distributions (Hickling, Roy, Hill, Fox, & Thomas, 2006), behaviors (Charmantier et al., 2008), and phenologies (Musolin, 2007; Musolin, Tougou, & Fujisaki, 2010; Parmesan, 2007) of various organisms, including insects (Thomson, Macfadyen, & Hoffmann, 2010; van Asch & Visser, 2007; Yang & Rudolf, 2010). Insects play important roles in natural and agroecosystems (Shaw & Hochberg, 2001), and understanding their responses to increased seasonal climate variation is necessary to inform future management and policymaking.

Outlining lethal and sublethal consequences for insects when temperatures approach their upper thermal limits should help to quantify risks of future losses in biodiversity (Hance, van Baaren, Vernon, & Boivin, 2006) and ecosystem productivity (Bonan, 2008). Upper thermal limits for insects are generally more rigid than lower thermal limits (Addo‐Bediako, Chown, & Gaston, 2000) and thus may be less affected by adaptive evolutionary change and phenotypic plasticity (Hoffmann, Chown, & Clusella‐Trullas, 2013; Kellermann et al., 2012). Host–parasitoid systems are among the most likely ecological interactions to be affected by increased climate variation (Visser & Both, 2005) due to the high trophic position of parasitoids and their tendency for host specialization, leading to the potential for disparity in phenological responses (Godfray, 1994) and range shifts (Davis, Jenkinson, Lawton, Shorrocks, & Wood, 1998).

Generally, hosts and parasitoids synchronize emergence via photoperiod (Beck, 2012) and temperature (Orr, Boethel, & Jones, 1985), and differing responses to variation in one of these cues could desynchronize local host and parasitoid populations (Stireman et al., 2005). Emergence times for host insects may also be differently affected by fluctuating thermal conditions provided by phenotypic variations. For instance, darker coloration of larvae can lead to increased body temperature and rate of development (Porter, 1983), and life history stages located in controlled environments (e.g., wood‐boring beetle larvae) are buffered from extreme environmental conditions influencing development of parasitoids and emergence times. Moving toward the equator, relative humidity becomes a more important factor in determining emergence times (Danforth, 1999; Seymour & Jones, 2000). However, while climate change currently impacts temperature disproportionately more in polar regions (Turner et al., 2014), the expanding range of these effects on ecological communities may lead to relative humidity becoming a more significant factor in disrupting phenological cycles.

Divergence in body size between parasitoids and hosts might play a role in thermal sensitivity (Campbell, Frazer, Gilbert, Gutierrez, & Mackauer, 1974; Walther et al., 2002) as well as dispersal rate (Roff, 1991), creating greater separation in phenology and potentially decreasing the overlap in their respective range limits (Davis et al., 1998). Furthermore, many parasitoids exhibit life history traits that make them prone to desynchronization with host species, including high host specificity and low dispersal rates (Jeffs & Lewis, 2013). For example, specialist parasitoids can be isolated from their hosts at geographic range‐margins because of the disparity in species‐specific (Jeffs & Lewis, 2013) and density‐dependent (McCann, Hastings, Harrison, & Wilson, 2000) dispersal rates, both of which have been shown to be impacted by extreme temperature events (Godfray, 1994). Thus, climate change could alter the synchrony of host–parasitoid phenologies and distributions, potentially releasing host species from parasitism. Although some research has examined how parasitoids will respond to colder temperatures (Klapwijk & Lewis, 2009) or experimental warming (Duan, Jennings, Williams, & Larson, 2014; Klapwijk, Gröbler, Ward, Wheeler, & Lewis, 2010), we still know relatively little about how increased climate variation could affect host–parasitoid interactions.

*Oobius agrili* Zhang and Huang (Hymenoptera: Encyrtidae) is a solitary egg parasitoid of emerald ash borer (EAB), *Agrilus planipennis* Fairmaire (Coleoptera: Buprestidae), a wood‐boring beetle that has become invasive in North America and Europe (Herms & McCullough, 2014; Orlova‐Bienkowskaja, 2014). Both *O*. *agrili* and EAB are native to northeastern Asia (i.e., China, the Korean Peninsula, and the Russian Far East), and *O*. *agrili* has been released for biological control of EAB in the United States since 2007 (Bauer, Duan, Gould, & Van Driesche, 2015). In most of North America, EAB adults emerge in late spring and early summer (Haack, Baranchikov, Bauer, & Poland, 2015). Adults are ready to mate after feeding on ash (*Fraxinus* spp.) leaves for around 1 week, with oviposition following around 1 week later (Cappaert, McCullough, Poland, & Siegert, 2005; Rodriguez‐Saona et al., 2007; Wang et al., 2010). EAB females oviposit eggs within cracks and crevices in the bark of ash trees, and individuals can produce around 40–70 eggs in a lifetime (Wei et al., 2007). EAB eggs typically hatch 2–3 weeks after oviposition (Wang et al., 2010), with larvae then burrowing through the cambium to feed on phloem. Larvae develop through four instars over summer and fall before chewing a pupal chamber, overwintering as mature larvae, and emerging as adults the following spring or summer; however, under some conditions EAB follow a semivoltine life cycle (Haack et al., 2015; Siegert et al., 2010).

The cryptic nature of EAB oviposition and the small size of adult *O*. *agrili* (~1 mm) make it impractical to study interactions between these species in the field. *Oobius agrili* has a quick generational turnover, typically characterized as a bivoltine or multivoltine seasonal cycle, as well as a parthenogenetic reproductive strategy. Wild population sex ratios have been documented at 29:1 (female:male) (Bauer & Liu, 2007), and current laboratory populations are entirely female biased (Larson & Duan, 2016), making it an ideal candidate for multigenerational lab experiments.

In this study, our objectives were to assess how experimentally controlled variation in climate affected host–parasitoid interactions between EAB and *O*. *agrili*. Specifically, for EAB we examined how climate variation affected their fecundity and survival, while separately for *O*. *agrili* we investigated how climate variation influenced their percent parasitism of EAB eggs, percent diapause, emergence times, and survival over successive generations when exposed to three different densities of EAB eggs. We predicted that increased temperature variation would add physiological stress for both host and parasitoid, thus correlating to a decrease in EAB fecundity and survival, and a decrease of both F_0_ and F_1_ *O*. *agrili* percent parasitism (independent of egg density), and survival. Temperature appears to have little effect on *O*. *agrili* diapause behavior (Hoban, Duan, & Hough‐Goldstein, 2016), but because changes in humidity can affect parasitoid diapause, we predicted that percent diapause would be influenced by humidity variation. Lastly, we hypothesized that increased temperature and humidity variability would disrupt larval development and therefore lead to greater variation in *O*. *agrili* F_1_ emergence times.

## 2 MATERIALS AND METHODS

### 2.1 Climate treatments

Four AR‐360 environmental chambers (Percival Scientific, Perry, IA, USA) were used for both experiments. Each chamber was assigned a specific climate variation treatment, which was monitored by a HOBO data logger (Onset Computer Co., Bourne, MA, USA) recording hourly temperature and relative humidity (RH) data (Table 1). Climate variation treatments were created by adjusting the increments in which temperatures increased/decreased each day, specifically: ±0°C (control), ±1°C (low), ±5°C (medium), and ±10°C (high). Thus, our aim was to keep the overall mean temperature in each chamber similar (~25°C) but generate a unique standard deviation (SD) in temperature. For example, in the medium treatment, over five consecutive days the daily temperature would cycle through 25°C, 30°C, 25°C, 20°C, and then 25°C. The upper and lower bounds of the temperature treatments (20°C and 30°C, respectively) were selected to be within future ranges predicted by climate models for northeastern United States (Kharin et al., 2007; Yao et al. 2013). We initially set RH to 65% for all chambers, but it was allowed to vary as a consequence of the specific temperature regime assigned to each chamber (Table 1). Historical data of daily temperature and humidity ranges and means from May–August in Maryland (National Weather Service 2015) were used to establish our control treatment. Maryland has a high density of EAB and several introduced parasitoid species, including *O*. *agrili* (Jennings, Duan, Larson, Lelito, & Shrewsbury, 2014; Jennings et al., 2016), making the location ecologically relevant.

**Table 1:** Environmental parameters in chambers for all four climate variation treatments. Monitoring was conducted hourly for the duration of the experiments (photoperiod, L:D = 15:9)

Treatment	Temperature (°C)				Relative humidity (%)			
	Mean	*SD*	Max.	Min.	Mean	*SD*	Max.	Min.
Control	23.98	1.24	25.56	19.81	70.34	2.81	89.40	48.20
Low	24.34	3.00	31.12	18.28	70.13	15.49	96.00	47.80
Medium	25.07	3.60	31.12	18.66	56.99	2.55	64.90	45.80
High	24.26	4.79	30.31	17.90	63.78	9.02	81.90	49.40

### 2.2 Eab response to climate variation

EAB used in this experiment (*n *=* *160) originated from naturally infested green ash (*Fraxinus pennsylvanica*) harvested in Prince George's County, Maryland. EAB adults were sexed and paired (one male and one female) and placed in 1‐L cups with fresh bouquets of tropical ash (*Fraxinus uhdei*). Black 1 × 1 mm nylon screen mesh lining and standard coffee filter paper covered the tops of these containers, which were secured with rubber bands. Coffee filter paper lined with mesh functions as an oviposition site for EAB females (Duan, Watt, Taylor, Larson, & Lelito, 2013). Replicates were placed into each environmental chamber (*n *=* *20 for each climate variation treatment), and adult survival was monitored daily. Bouquets and coffee filter paper were replaced semi‐weekly, and EAB fecundity and survival (days) were recorded.

We examined how EAB fecundity was affected by temperature variation, humidity variation, and their interaction, using a general linear model. To improve normality, counts of eggs were square‐root transformed. EAB survival in relation to climate variation treatment was investigated using Kaplan–Meier survival analysis, and assessed for significance using log‐rank tests. All analyzes were conducted using R 3.3.2 (R Core Team 2016).

### 2.3 *Oobius agrili* response to climate and host density variations

All F_0_ *O*. *agrili* used in this experiment (*n *=* *120) were taken out of chill (1.7°C) and placed in 25°C to emerge from diapause. This procedure mimics the transition from winter to spring and cues the parasitoid to emerge (Larson & Duan, 2016). Once emerged, *O*. *agrili* adults were placed in snap‐cap vials with adequate ventilation. Honey was applied to vials ad libitum for the duration of the experiment.

**Figure 1:**
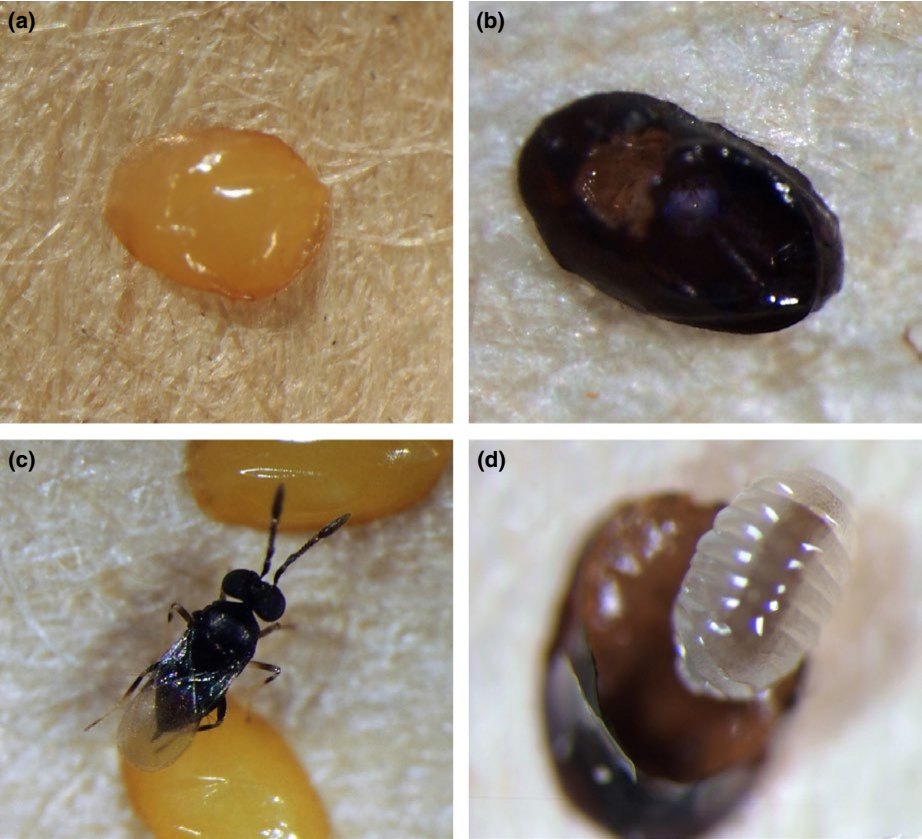
Different fates of *Oobius agrili* eggs: (a) unparasitized, (b) parasitized, (c) nondiapaused (i.e., emerged adult), and (d) diapaused. Photo credit: Kristi M. Larson (United States Department of Agriculture, Agricultural Research Service)

We used a 4 × 3 experimental design (four climate variation treatments crossed with three EAB egg densities) to assess the effects of climate variation on *O*. *agrili*. Individuals were designated an environmental chamber (Table 1) and weekly host egg density treatment (6, 12, or 24 eggs) for the remainder of the study. Occasionally, unfertilized eggs were inadvertently included in parasitoid exposures, so there was a small amount of variation in the egg density treatments. EAB eggs were laid at optimal environmental conditions (25°C, 65 ± 10% RH, L:D = 15:9), and within 48 hours they were exposed to the parasitoid (48‐hr exposure; L:D = 30:18). Once exposures were completed, exposed eggs were removed from the snap‐cap vials and placed in prehoneyed, labeled screw‐cap vials. Screw‐cap vials were monitored daily for parasitoid emergence. Progeny F_1_ that emerged during this experiment (*n *=* *160) were designated to the same environmental chamber and underwent the same weekly exposures as parents *F*_0_. Exposed eggs were dissected under an Olympus SZH microscope (Olympus Inc., Center Valley, PA, USA) 7 weeks after the initial exposure date. This time ensures that all exposed eggs could be correctly identified as either unparasitized (Figure 1a) or parasitized (Figure 1b). Additionally, by this time, all parasitized eggs had either emerged as adults (nondiapaused *O. agrili*; Figure 1c) or had developed into their overwintering stage (diapaused *O. agrili*; Figure 1d). Data quantified for *O*. *agrili* included the following: percent parasitism of EAB eggs, percent diapause, emergence time (days), and survival (days).

We first investigated how *O*. *agrili* percent parasitism and diapause were affected by temperature variation, humidity variation, generation, and host egg density using generalized linear models with quasi‐binomial error distributions (Crawley, 2012). We then examined how *O*. *agrili* emergence was affected by temperature variation and humidity variation using a generalized linear model with a Gaussian error distribution. Model goodness‐of‐fit was tested using chi‐square tests based on model deviance and residual degrees of freedom (*df*). Significance was then assessed using type II sums of squares. All two‐way interactions were tested, and nonsignificant interactions (*p *>* *0.05) were dropped from the final models. *Oobius agrili* survival in relation to climate variation treatment was investigated using Kaplan–Meier survival analysis, and significance was assessed using log‐rank tests. All analyzes were conducted using R 3.3.2 (R Core Team 2016).

## 3 RESULTS

### 3.1 Eab response to climate variation

**Figure 2:**
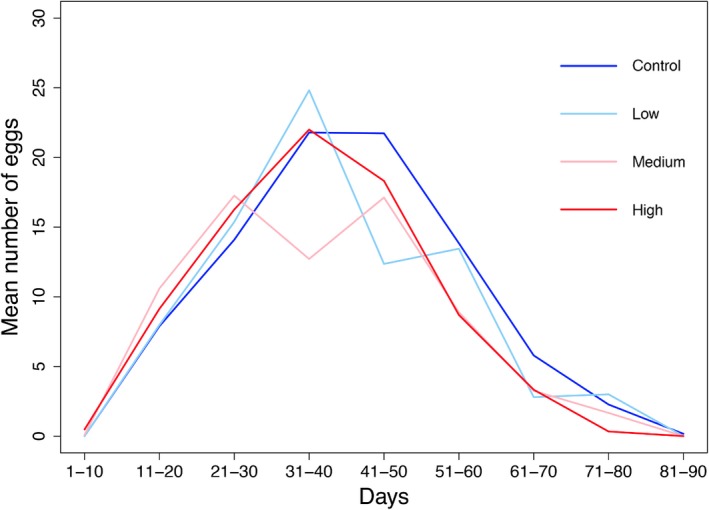
Mean number of eggs per emerald ash borer in different climate variation treatments over time. Standard deviations in temperature for climate variation treatments are as follows: control = 1.24°C, low = 3.00°C, medium = 3.60°C, and high = 4.79°C

EAB fecundity was negatively associated with temperature variation (*F*_1,76_ = 9.52, *p *=* *0.003), with a mean number of eggs in the control treatment more than double that produced in the high variation treatment (mean number of eggs ± *SE*: control = 206.00 ± 26.4, low = 108.65 ± 29.71, medium = 75.9 ± 26.77, high = 92.50 ± 28.16). Furthermore, under the highest temperature variation treatment the EAB oviposition period shifted temporally (Figure 2). More specifically, EAB females under the highest temperature treatment started producing eggs 4 days earlier than the controls, and stopped producing eggs 10 days before the controls. We did not detect any significant effects of humidity variation (*F*_1,76_ = 0.06, *p *=* *0.802) or the interaction between temperature and humidity (*F*_1,76_ = 3.48, *p *=* *0.066) on EAB fecundity.

**Figure 3:**
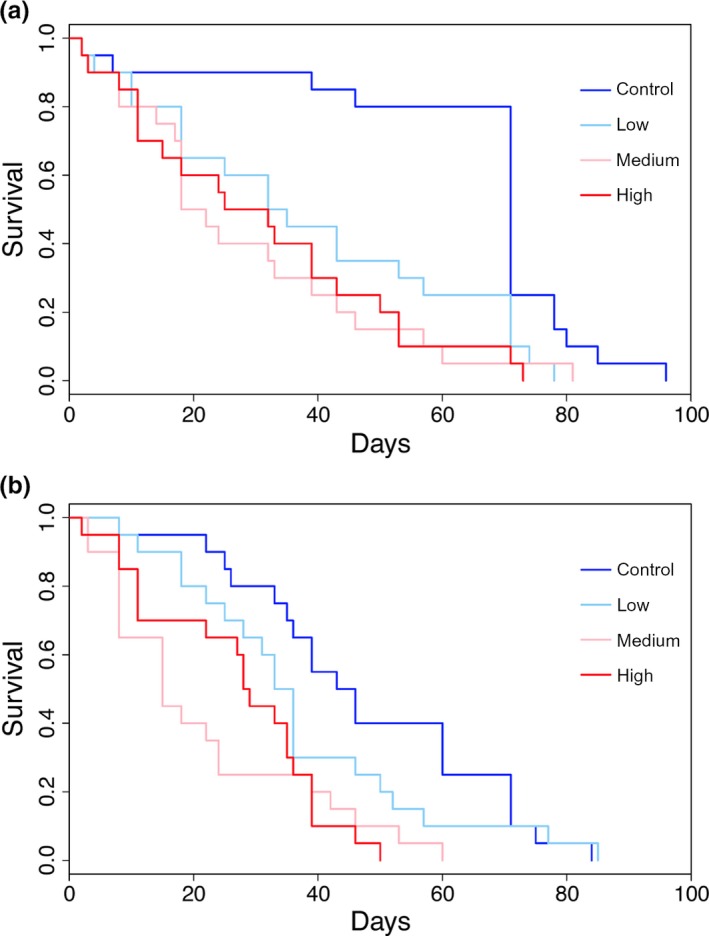
Female (a) and male (b) emerald ash borer survival in different climate variation treatments over time. Standard deviations in temperature for climate variation treatments are as follows: control = 1.24°C, low = 3.00°C, medium = 3.60°C, and high = 4.79°C

Survival for both sexes differed significantly by climate variation treatment (females: log‐rank = 23.05, *df* = 3, *p *<* *0.001, males: log‐rank = 17.00, *df* = 3, *p *<* *0.001), but generally survival was higher in treatments with lower variation in climate (Figure 3).

### 3.2 *Oobius agrili* response to climate and host density variations

**Figure 4:**
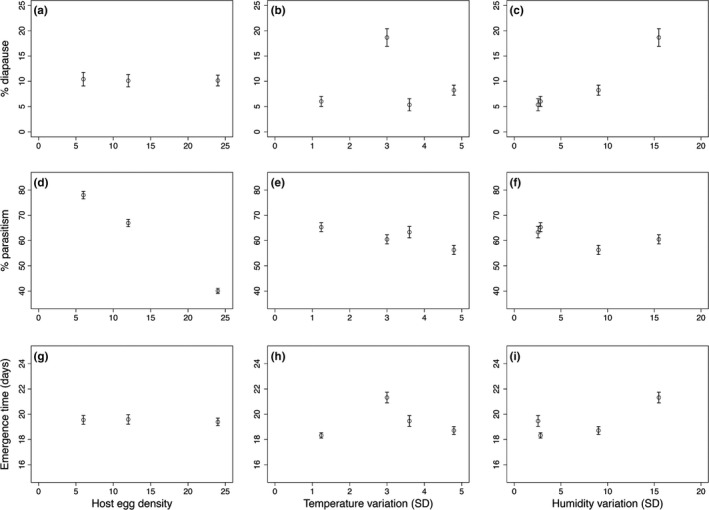
Behavioral responses of *Oobius agrili* to changes in host egg density, temperature variation (°C), and humidity variation (RH). Shown are effects on percent diapause (a, b, c), percent parasitism (d, e, f) and emergence times (g, h, i)

We found that parasitoid diapause was positively associated with humidity variation, with 18.67% of both generations of *O*. *agrili* entering diapause under the highest humidity variation treatment compared with 5.35% when humidity variation was lowest (Table 2; Figure 4c). Parasitoid generation also significantly affected diapause in all experimental treatments, with over six times more diapaused individuals in the *F*_1_ generation (17.59 ± 1.20%) compared with the *F*_0_ generation (2.74 ± 0.47%; Table 2). However, there were no significant effects of temperature variation (Table 2; Figure 4b) or host egg density (Table 2; Figure 4a) on *O*. *agrili* diapause.

**Table 2:** Results from generalized linear models testing effects of generation, host egg density, and climate variation treatments, on *Oobius agrili* diapause and parasitism (quasi‐binomial error distributions), and emergence times (Gaussian error distribution)

Effect	Diapause (%)	Parasitism (%)	Emergence (days)
Generation	**190.58 (<0.001)**	**21.13 (<0.001)**	–
Host egg density	0.89 (0.344)	**438.86 (<0.001)**	0.64 (0.424)
Humidity variation	**92.68 (<0.001)**	0.04 (0.845)	**36.36 (<0.001)**
Temperature variation	2.38 (0.123)	**4.86 (0.028)**	1.47 (0.226)
Generation × Host egg density	0.03 (0.859)	0.05 (0.824)	–
Generation × Humidity variation	2.72 (0.099)	0.04 (0.838)	–
Generation × Temperature variation	1.27 (0.260)	3.48 (0.062)	–
Host egg density × Humidity variation	0.04 (0.846)	0.04 (0.833)	<0.01 (0.976)
Host egg density × Temperature variation	0.13 (0.716)	2.48 (0.115)	0.25 (0.617)
Humidity variation × Temperature variation	0.02 (0.899)	0.74 (0.389)	**13.69 (<0.001)**

Note that interactions with *p *>* *0.05 were dropped from the final models, and all *df* = 1. Statistically significant effects (*p* < 0.05) are denoted in bold.

*Oobius agrili* percent parasitism declined with EAB egg density from 77.98% with six host eggs to 40.07% with 24 host eggs (Table 2; Figure 4d), most likely reflecting the maximum threshold for parasitoid egg production between exposure events (48 hr). Parasitism also was significantly higher in the *F*_1_ generation (63.55 ± 1.32%) compared with the *F*_0_ generation (58.94 ± 1.35%; Table 2). Finally, although temperature variation appeared to affect parasitism in a nonlinear way, the relationship between them generally was negative (Table 2; Figure 4e). There was no effect of humidity variation on parasitism (Table 2; Figure 4f).

Temperature variation (Table 2; Figure 4h) and humidity variation (Table 2; Figure 4i) interacted to affect parasitoid emergence. Specifically, under lower temperature variation treatments, *O*. *agrili* emergence times were positively associated with humidity variation, while under higher temperature variation treatments the relationship was negative. Emergence times peaked during the low temperature variation treatment and were ~2–3 days longer than for any of the other treatments. Host egg density had no effect on emergence times (Table 2; Figure 4g).

**Figure 5:**
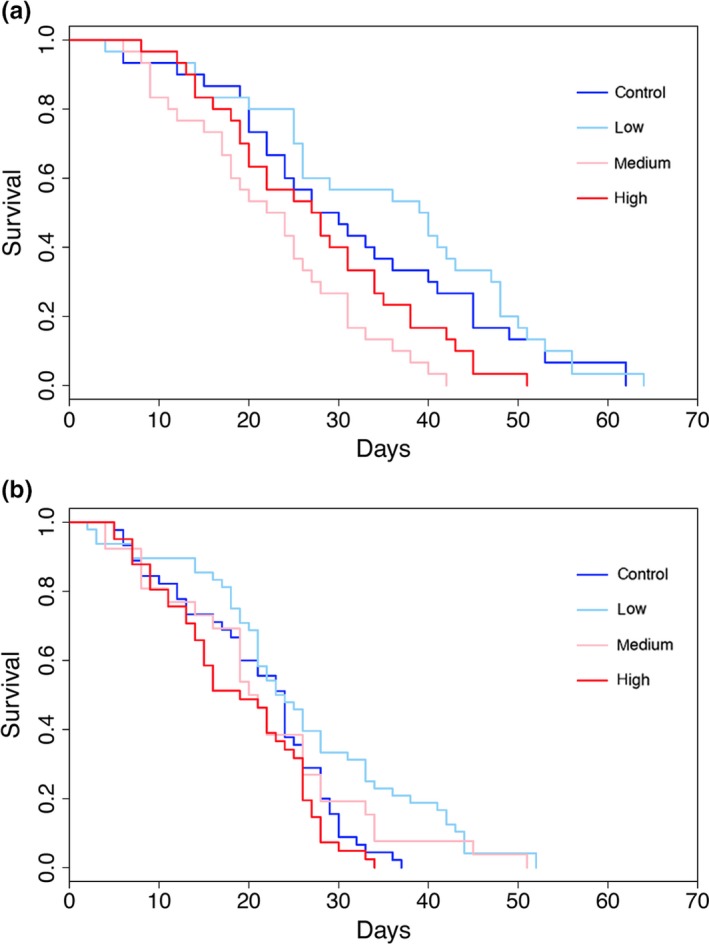
F0 (a) and F1 (b) *Oobius agrili* survival in different climate variation treatments over time. Standard deviations in temperature for climate variation treatments are: control = 1.24°C, low = 3.00°C, medium = 3.60°C, and high = 4.79°C

*Oobius agrili* survival for both generations was significantly influenced by climate variation treatment (*F*_0_: log‐rank = 20.66, *df* = 3, *p *<* *0.001, *F*_1_: log‐rank = 13.16, *df* = 3, *p *=* *0.004). While survival for the F_0_ generation was generally highest in the lower climate variation treatments, in the *F*_1_ generation survival was low in the control treatment (Figure 5).

## 4 DISCUSSION

Findings from the present study supported our hypothesis that climate variation, particularly in terms of temperature, can negatively affect both EAB fecundity and survival. Our results further showed that EAB initiated oviposition earlier and had a shorter oviposition period when climate was more variable. The earlier initiation and shorter duration of oviposition associated with increased temperature variation may represent the “egg‐dumping” behavior of female EAB. Such “egg‐dumping” behavior has also been observed with other beetles such as *Callosobruchus maculates* when stressed with abnormal conditions such as host‐deprivation and/or food‐shortages (e.g., Messina & Fox, 2011; Messina, Morrey, & Mendenhall, 2007). In addition, our study showed that both *O*. *agrili* survival and parasitism rate generally exhibited a negative response to climate variation as well as host densities. Together, these findings suggest that increased frequencies of extreme heat waves accompanying climate change could greatly disrupt the host utilization and synchronization behaviors of parasitoids.

Previous work by Duan et al. (2014) examined the relationship between EAB and *O*. *agrili* under constant temperature treatments (20–35°C, at increments of 5°C) and found significantly longer emergence times for *O*. *agrili* at 20°C (38 days) compared with all other treatments, and no significant difference in emergence time between 25°C (18 days) and 30°C (17 days). Additionally, Duan et al. (2014) found the duration of time for host egg susceptibility to parasitism decreased at higher mean temperatures, which could have an impact on parasitoid search time and efficiency as seasonal temperature extremes continue to increase in frequency and severity. In the present study, manipulating the variance but maintaining mean temperature significantly impacted emergence times nonlinearly. It is important to note that the time‐frame of parasitism susceptibility used by Duan et al. (2014) was longer than the exposure time in our experiment so this would have had a negligible effect on the mean parasitism values we observed.

The rates of *O*. *agrili* diapause we observed were generally within the ranges found elsewhere (Hoban et al., 2016; Larson & Duan, 2016), albeit under different environmental conditions. Diapause and emergence times in some insects are known to be affected by humidity (Danforth, 1999; Seymour & Jones, 2000). Indeed, Hoban et al. (2016) showed that photoperiod was a major determinant of *O*. *agrili* diapause. However, with photoperiod constant across all treatments in our experimental design, our findings suggest that relative humidity could also be an important factor for initiating diapause in these parasitoids. These results were somewhat surprising because photoperiod and temperature generally are the most important factors affecting diapause termination in temperate insects like *O*. *agrili* (Tauber, Tauber, & Masaki, 1986), while humidity may be more critical for tropical species (Seymour & Jones, 2000). Another variable affecting *O*. *agrili* diapause and emergence times could be resource availability (Canzano, Jones, & Seymour, 2003), although we did not manipulate resources in the present study.

The physiological response by *O*. *agrili* may be explained by both the nonlinearity of insect thermal performance curves, which have been extensively studied with respect to many fitness parameters (Angilletta, 2009), and the differing timescales of recovery from cold and heat stress due to distinct physiological constraints (Roitberg & Mangel, 2016). For example, a significantly longer recovery time of *Drosophila* from heat comas as opposed to chill comas can be attributed to a differentiation in response time of heat and cold‐shock proteins (Goto & Masahito, 1998). Furthermore, contrasting responses to extreme temperature can arise within different life history events. A recent study observing the response of aphids to laboratory‐simulated heat waves showed significant differences in adult fecundity and survival but no change in development time (Ma, Volker, & Chun‐sen, 2015). Therefore, a dissimilarity between emergence time and parasitism rate (also to a lesser extent diapause strategy in *F*_1_ *O*. *agrili*) could be reasoned within this context.

Variance in temperature adds complexity to the relationship between an organism's performance and the resulting population dynamics (Kingsolver, Higgins, & Augustine, 2015; Kingsolver, Woods, & Woods, 2016; Roitberg & Mangel, 2016). Recently, Estay, Lima, and Bozinovic (2014) examined the separate and combined impacts of increasing thermal mean and variability on population stability and showed that the point of criticality along the performance curve (where the net effect went from positive to negative) was at the inflection point for their increasing thermal variability scenario. These findings have potential implications for the performance of *O*. *agrili* at or beyond their inflection points, which may not be synchronized with their host species. For instance, when experiencing conditions of high temperature variation, EAB switched to the “egg‐dumping” behavior described previously, and *O*. *agrili* percent parasitism on those eggs was reduced. Thus, greater variation in climate may initially be most detrimental to populations of *O*. *agrili*.

Similar to Estay et al. (2014), our findings highlight the importance of incorporating elements of ecophysiology and population ecology into research on the ecological effects of climate change. An alternative theoretical approach focusing instead on generic foraging traits (such as detection distance, search rate, and handling time) was implemented by Dell, Pawar, and Savage (2014) to study the impact of temperature change on consumer–resource interactions and produced qualitatively similar results to that of Estay et al. (2014). Further, again in agreement with Estay et al. (2014), Dell et al. (2014) elucidate the importance of asymmetry between optimal thermal conditions for consumers and resources as a prerequisite for an uncoupling between species. These two theoretical approaches emphasize generic routes for temperature variation to significantly impact the stability of host–parasitoid interactions.

The present study adds to the growing literature indicating that ectotherm species‐specific responses to climate variation are not always intuitive and that they can potentially have broad ecological effects (Paaijmans et al., 2013; Rohr & Raffel, 2010). Specifically, we hope to emphasize the impact that climate variability can play in consumer–resource interactions, either via differing responses toward the same environmental cue (e.g., ambient temperature variation) or a decoupling of multiple environmental cues (e.g., ambient temperature and humidity variation). Decoupling of these cues may act as a driver for temporal isolation of the resource species, resulting in future spatial fragmentation between consumer and resource populations. Further empirical and spatially explicit theoretical studies examining generic consumer–resource interactions should help to elucidate the broader, community‐based responses to climate change (Gilman, Urban, Tewksbury, Gilchrist, & Holt, 2010).

Our findings also have more specific implications for arthropod biological control. For instance, introduced parasitoids could become less effective at controlling target organisms because of asynchrony in emergence times, leading to increases in the frequency and intensity of pest outbreaks (Stireman et al., 2005). Accounting for the declines in parasitism rate, host egg susceptibility to parasitism and time‐frame of host egg production as functions of temperature and humidity, natural populations of *O*. *agrili* (in particular the *F*_1_ generation emerging in summer), may be at risk of temporal isolation from host resources upon emergence as climate change induced seasonal temperature variation magnifies. Furthermore, as the disparity between‐population fronts of hosts and parasitoids increases due to temporal isolation, parasitoid life history traits such as dispersal rate and host specialization will become more important in predicting the success of future biological control programs. Consequently, incorporating predicted changes in climate into future biological control programs should be considered in terms of potential environmental impact and risk assessment (Wu, Hoffmann, & Thomson, 2016). This approach will also aid in managing current biological control programs, for example by enabling us to predict under what environmental conditions host phenological and geographical shifts might occur. Phenotypic and phenological plasticity of parasitoids is widely known to allow for stability under certain environmental conditions (see Hance et al., 2006). However, beyond some threshold these attributes are no longer sufficient for maintaining stable dynamics, and decoupling is inevitable.

Several limitations to the present study prevent us from drawing broader conclusions regarding the influence of climate variation on host–parasitoid interactions but provide opportunities for further research. For instance, our experiments largely examined the effects on hosts and parasitoids separately, meaning that the potential effects of different timings for host fecundity and parasitoid emergence may not ultimately lead to asynchrony, though using identical environmental conditions for both host and parasitoid treatments allow us to draw result‐driven conclusions. Logistically, it is challenging to monitor the survival and behaviors of a parasitoid as small as *O*. *agrili* within the larger and more complex environment necessary to maintain EAB. However, if possible, doing so would undoubtedly make the research more ecologically relevant. Longer term experiments in larger mesocosms would also enable us to investigate if climate variation affects host phenology and voltinism, in addition to fecundity. Finally, we did not manipulate host plant resources in the present study. Changes in temperature and precipitation can greatly affect host plants in several ways, such as their biomass, chemical composition, defenses, and nitrogen content (Jamieson, Trowbridge, Raffa, & Lindroth, 2012; Wade, Karley, Johnson, & Hartley, 2017), as well as their geographic distribution (Liang & Fei, 2014). Subjecting host plants to the same environmental conditions as the herbivores and parasitoids would facilitate greater understanding of multitrophic interactions. Consequently, while our results suggest that climate variation can affect host–parasitoid synchrony, these findings are only a step toward increasing our understanding of how changes in temperature and humidity affect species interactions.
